# Acceptance of the ‘Assessment of Physiotherapy Practice (Chinese)’ as a standardised evaluation of professional competency in Chinese physiotherapy students: an observational study

**DOI:** 10.1186/s12909-020-02026-3

**Published:** 2020-04-09

**Authors:** Jia Hu, Alice Y.M. Jones, Xuelian Zhou, Hua Zhai, Shirley P. C. Ngai, Ka-Chun Siu, Megan Dalton

**Affiliations:** 1 Shanghai Yangzhi Rehabilitation Hospital (Shanghai Sunshine Rehabilitation Center), Tongji University School of Medicine, Shanghai, China; 2 School of Health and Rehabilitation Sciences, The University of Queensland, Brisbane, Australia; 3 Faculty of Health Sciences, The University of Sydney, Sydney, Australia; 4 Department of Rehabilitation Sciences, The Hong Kong Polytechnic University, Kowloon, Hong Kong, China; 5 Physical Therapy Education, University of Nebraska Medical Center, Omaha, NE USA; 6 School of Allied Health, Australian Catholic University, Brisbane, Australia

**Keywords:** Physiotherapy practice assessment, Clinical education, Competency-oriented assessment tool

## Abstract

**Background:**

Development of an entry-level physiotherapy curriculum in China currently follows the World Confederation for Physical Therapy (WCPT) guidelines, however there is no standard, validated, assessment tool for physiotherapy practice in use in China. This article reports the process of translation of the “Assessment of Physiotherapy Practice” (APP), a validated assessment instrument adopted by all universities in Australia and New Zealand, into Chinese (APP-Chinese) and its implementation by Chinese physiotherapy clinical educators (CEs) and students during clinical placements.

**Methods:**

The process of forward and backward translation of the APP was undertaken by a team of academics from universities in Shanghai, Hong Kong, United States and Australia. An APP-Chinese version was produced and used for assessment of the clinical performance of 4th year students at a university in Shanghai. Feedback on the implementation of the APP-Chinese was solicited from students and CEs using the same two questionnaires employed to assess implementation of the original APP.

**Results:**

All CEs agreed that the rules used to score the APP-Chinese were helpful in assessing student performance. Over 90% of the CEs considered the APP-Chinese was pragmatic for use in the clinical environment in China. All students agreed with the rating of their performance on the APP-Chinese marked by their educators, and that the performance indicators were useful in guiding their expected performance behaviour.

**Conclusion:**

The APP-Chinese is the first standardised assessment tool for evaluation of clinical performance of physiotherapy students in China and was shown to be well accepted by both students and CEs in the clinical education unit and university involved in this study.

## Background

The health-care system in China has evolved from both traditional Chinese and Western medicine. Physiotherapy is still not as yet recognised by the Chinese Government as a profession. Current rehabilitation services in China are led by physicians in rehabilitation medicine assisted by rehabilitation therapists. Rehabilitation therapists can be trained through a 4-year bachelor program or a 3-year diploma program, with a curriculum based on medical management and an introduction to treatment techniques used by physiotherapists, occupation therapists, speech therapists and traditional Chinese doctors. The first structured evidence-informed physiotherapy curriculum that meets international standards was a master entry-level program jointly organised by the Hong Kong Polytechnic University and Tongji Medical College of Huazhong University of Science Technology in Wuhan in 2004.

The 2004 program marked the beginning of an independent, entry-level physiotherapy curriculum. To date, there are 6 entry-level physiotherapy programs in China that have been accredited by the WCPT [1]. However, due to a lack of experienced physiotherapists, physiotherapy practice in the clinical environment is still in the developmental stage. Clinical education is a fundamental process assisting students to apply theoretical knowledge to clinical practice [2, 3]. Clinical education provides an integral environment which allows students the opportunity to learn how to employ clinical reasoning and evidence-based practice in patient management [4]. There is no standardised level of practice competence for physiotherapy in China. Unlike Australia and New Zealand and many western countries, assessment of the clinical performance of physiotherapy students in China is not based on standardised practice guidelines. The existing assessment of physiotherapy students during Chinese clinical placement is varied amongst different hospitals, and may include theoretical examination, operational skill examination, report writing or case reporting [5–8]. There are no standardised assessment tools with appropriate evidence of validity and reliability.

Three professional practice assessment forms are currently recommended by the WCPT guidelines for Clinical Education [9]. These forms are the American physical therapist clinical performance instrument (PT-CPI) [10], the Australian Assessment of Physiotherapy Practice (APP) [11, 12], and Ireland’s Common Assessment Form (CAF) [13]. There is considerable commonality in the areas of practice evaluated by these instruments, with aspects of patient assessment and management, professional behavior and communication included in these instruments. A key difference between these instruments relates to the use of Rasch analysis as the measurement model used during development and evaluation of the psychometric properties of the tool [14–17]. A critical advantage of Rasch measurement is that it enables the abstraction of equal units of measurement from the raw data of observations, i.e. scores on the items of an assessment tool. These can be calibrated and then used with confidence to measure and quantify human attributes such as competency in physiotherapy practice [18]. This conversion facilitates appropriate interpretation of differences between individuals and allows tallying of scores to provide a meaningful total score. In addition, Rasch analysis enables testing of the internal construct validity of a scale for uni-dimensionality (considered an essential quality of a scale scored by adding results of items) and identification of gaps in the targeting of items to the students’ abilities. Rasch analysis also enables assessment of item bias through investigation of differential item functioning. Of the three instruments recommended by WCPT, the APP employed Rasch analysis in the development and evaluation of the instrument [11]. Evidence of structural validity supported the interpretation that a student’s score on the APP is an indication of their underlying level of professional competence as demonstrated during clinical placements.

The APP is used as a standardized assessment tool for the clinical practice of physiotherapy students in Australia and New Zealand since 2011 [11]. The aim of this study was to translate the original APP into Chinese and investigate the acceptance of the Chinese version of APP as a standardised clinical performance evaluation tool by Chinese physiotherapy students and educators in a pilot University. Should this be shown to be accepted by physiotherapy students and CEs in the pilot University, the next step would be to conduct a full-scale validity study on the Chinese version of the APP and introduce the validated version as a standardised assessment instrument for clinical performance of all physiotherapy students in China.

## Methods

### Design

This is an observational study describing Chinese physiotherapy student’s perception of using the translated APP-Chinese for evaluation of their clinical performance. Approval to conduct this study was obtained from the Hospital Ethics Committee of Shanghai Yangzhi Rehabilitation Hospital (Shanghai Rehabilitation Center). (Ethics approval number: YZ2017–003). The protocol adopted in this study is available at protocol.io: dx.doi.org/10.17504/protocols.io.598g99w.

### Participants and setting

Participants in this study included the 4th year Physiotherapy students at the Shanghai Tongji University. The physiotherapy program at the Shanghai Tongji University is accredited by the WCPT. Clinical educators (CEs) involved in this study were qualified physiotherapists at the Shanghai Sunshine Rehabilitation Center, an affiliated hospital of Tongi University.

### Translation of the APP

Translation of the original APP was undertaken by following the process of ‘establish expert committee, forward translation, backward translation’ as described by Tsang et al. 2017 [19].

#### Forward translation

Two physiotherapy academic staff (from the university in Shanghai HJ & from the university in America, SKC) who received their education in China and English-speaking countries translated the English APP (Additional file 1) into Mandarin Chinese with Simplified Chinese characters (Chinese version 1). This version was then reviewed by a third bilingual physiotherapy educator, AJ, with teaching experience in both China and Australia, as well as research experience in education. Any discrepancies or minor modifications to the wordings and terminology proposed by AJ were discussed by the 3 educators and an agreed Chinese version 2 was developed.

#### Backward translation

An experienced physiotherapy academic from Hong Kong, SN, fluent in both English and Chinese languages and with publishing experience in both languages was invited to translate the Chinese version 2 back into English. SN was blinded to the English version of the APP and Chinese version 1 prior to her participation in this project. The outcome of this translation was an English Version 3.

Version 3 was then sent to MD, the original author of APP for comparison with the original English version. A Skype meeting was held between AJ and MD, to discuss the significance of any wording differences between the two English versions. A meeting was held with all 5 educators for ratification of any modifications deemed necessary. The final version (APP-Chinese) (Additional file 2) was produced to be piloted by physiotherapy students of the Shanghai Tongji University and CEs from the Sunshine Rehabilitation Center in Shanghai, China.

### Implementation of the APP-Chinese

All CEs and students involved in this project were invited to sign a consent form prior to the clinical placement.

#### Preparation of the CEs

Fourteen CEs from the Sunshine Rehabilitation Centre affiliated to the involved university were invited to attend a workshop conducted by AJ to introduce the APP-Chinese. The content of the workshop was based on similar introductory workshops conducted for CEs in Australia by the original author of the English APP. All CEs were provided with information on scoring rules associated with the APP-Chinese assessment form and examples of behavioral performance indicators extracted and translated from the English APP manual. Video vignettes of varying levels of student performance covering inadequate, adequate and excellent standards were used to facilitate calibration of assessor expectations and achieve consensus moderation.

#### Preparation of the students

Fifteen 4th year physiotherapy students at the involved university, undertaking three 8-week clinical placements (in cardiopulmonary, neurological and musculoskeletal physiotherapy subspecialties) at the Shanghai Sunshine Rehabilitation Centre, between July 2017 and February 2018, consented to participate in the study. Prior to the clinical placements, students were introduced to the APP-Chinese and the associated performance/ behaviour indicators were explained. A mid-unit feedback was organised for each student during the 4th week of each placement. Similarly, an end-unit feedback was organised at the end of 8th week of each placement.

### Post-placement feedback on the use of APP-Chinese

After three rounds of clinical placements, students and CEs were asked to complete the respective feedback questionnaires on the use of the APP-Chinese. These questionnaires were translated from the English APP feedback questionnaire used during the development and evaluation of the APP [20].

To explore views on the use of the APP-Chinese that might not be reflected by the standard APP questionnaire, a semi-structured focus group discussion session was separately organised for students and CEs after completion of the feedback questionnaires. The feedback sessions were led by investigator HJ. The group meeting focused on the following: comments on the 20-items in APP-Chinese and on the ease of understanding the wordings used in the APP-Chinese; applicability of the suggested examples of performance indicators for the Chinese clinical education environment; and any further comment regarding this method of assessment of clinical placement performance (using the APP-Chinese).

### Data analysis

Feedback data were entered into an excel data sheet and statistical analyses were performed using IBM® SPSS Statistics V.23.0 (IBM Corp. in Armonk, NY). Descriptive statistics were used to report age, clinical education years, completion time, and the score of each item. These values were expressed as mean ± standard deviation (SD). Students’ responses on the 5-point Likert scale feedback questionnaires (1 = Strongly disagree, 2 = Disagree, 3 = Uncertain, 4 = Agree, 5 = Highly agree) were reported as percentage of agreement for each statement. Students who scored 4 and 5 were grouped into ‘agreed’ category and those who scored 1, 2, or 3 were grouped into disagreed category.

## Results

A total of 14 CEs consented to participate in this pilot study. The male to female CE ratio was 8:6; average age of the CEs was 29.43 ± 1.91 years. The average experience of the CEs in physiotherapy was 5.36 ± 1.94 years. All CE participants were at senior physiotherapy level and 8 had a Masters degree in Physiotherapy. A total of 15 students, including 2 males, participated in this study. The mean age was 22.20 ± 0.68 years old. All students completed three 8-week sub-specialty placements.

### Feedback from clinical educators on the use of APP-Chinese

All 14 CEs returned the feedback form. A majority agreed that they could confidently use both the item rating scale (85.7%) and the global rating scale (92.9%) to evaluate the student performance. The reported completion time was 9.64 ± 7.1 min. All agreed that the scoring rules are helpful (100%) and performance indicators were clear and easy to understand (78.6%). About 71% of the CEs considered that the level of expected competency of an entry-level physiotherapist (passing level) was clear to them. The mean score of each feedback items reported from our CEs was found to be very similar to that reported by Australian and Canadian CEs [20] (Table 1).

**Table 1:** Mean score of the feedback items from Chinese clinical educators using the APP-Chinese compared to reported scores from Australian clinical educators using the APP [10]

Feedback items	Percentage of agreement	^−^Mean ± SD (China)	^−^Mean ± SD (Australia)
Confident using the 0–4 rating scale	85.7%	3.86 ± 0.36	3.96 ± 0.66
Confident using the global rating scale	92.9%	3.93 ± 0.27	4.00 ± 0.76
Performance indicators are useful	85.7%	4.07 ± 0.62	4.10 ± 0.70
Scoring rules are helpful	100%	4.21 ± 0.43	4.10 ± 0.70
The level of competency expected of an entry-level physiotherapist is helpful	71.4%	4.00 ± 0.78	4.00 ± 0.80
Performance indicators are easy to understand	78.6%	3.93 ± 0.62	4.10 ± 0.60
The APP is practical in clinical environment	92.9%	4.07 ± 0.48	4.10 ± 0.60
Time taken to complete the APP is acceptable	85.7%	3.79 ± 0.58	4.20 ± 0.70
Information on how to complete the APP-Chinese is comprehensive	85.7%	4.00 ± 0.56	4.20 ± 0.70
Preference of online version	85.7%	4.07 ± 0.62	3.30 ± 1.00

*SD* Standard deviation

### Feedback from students

A total of 15 completed feedback forms were received. All students agreed that: their CEs could correctly evaluate the 20 performance indicators on the 0–4 point scale of the APP-Chinese; and that the global rating scale reflected their performance in a fair manner. All agreed that performance indicators associated with the APP-Chinese were useful to guide behavioural expectations and the scoring rules were appropriate. However, only 66% of students agreed that the level of expected competency criteria for an entry-level physiotherapist (passing level) was clear to them. The feedback score for each item was comparable to that reported by Australian students (Table 2).

**Table 2:** Mean score of the feedback items from Chinese students using the APP-Chinese compared to reported scores from Australian students using the APP [10]

Feedback item	Percentage of agreement	^−^Mean ± SD (China)	^−^Mean ± SD (Australia)
Clinical educators can correctly use the APP-Chinese with a 0–4 point rating scale	100%	4.20 ± 0.41	4.10 ± 1.00
Performance indicators are useful for evaluating the performance before mid-term feedback	100%	4.13 ± 0.35	3.90 ± 0.70
The scoring rules are appropriate	100%	4.33 ± 0.49	4.00 ± 0.90
The expected entry-level performance (passing level) was clear to me	66%	3.67 ± 0.49	4.00 ± 0.90
Easy to understand	93%	3.93 ± 0.26	3.90 ± 0.70
Practical in clinical teaching	80%	3.93 ± 0.59	4.10 ± 0.70
Understand the level of performance corresponding to score of ‘4’	86.7%	4.07 ± 0.59	3.90 ± 1.00
Received sufficient information on the APP before the start of the clinical placement	86.7%	4.00 ± 0.54	4.10 ± 0.70
The global rating scale (GRS)can reflect my performance fairly	100%	4.13 ± 0.35	4.00 ± 1.30
Each of the 20 indicators can reflect my performance fairly	86.7%	4.13 ± 0.64	3.90 ± 1.20

*SD* Standard deviation

### Semi-structured interview

All 14 CEs attended a discussion session (independent from students’ group). All CEs agreed that the wordings used in the APP-Chinese and the instrument was considered clear and easy to understand. Additional comments included preference for a mobile version of the assessment form, and the difficulty of assessing interprofessional communication on patient management because students in a Chinese clinical environment are not permitted to communicate directly with other health care professionals regarding patient management.

All 15 students attended the semi-structured interview: There were no additional comments regarding items on the APP-Chinese. All agreed the wordings on the APP-Chinese were clear and easy to understand. Other comments included: appreciation of the open attitude of CEs during formative feedback sessions; the performance indicator examples were helpful; and use of the APP to develop strategies for improvement of performance were rated highly.

## Discussion

Clinical education is an essential element of an entry-level physiotherapy education program [9]. A well-structured clinical education program provides opportunities for students to integrate knowledge, skills and professional behaviours and apply them in a clinical setting including hospitals, clinics, as well as primary health care and community settings [9]. The ultimate aim of a clinical education program is to assist students to develop behaviours and skills that are requisites of the profession and that the student becomes a competent and autonomously practicing entry-level practitioner. Valid and reliable assessment tools are, therefore, essential to ensure the appropriate and expected outcomes are measured. The majority of clinical centres in China provide a summative feedback to students at the end of a clinical placement and most universities only require a pass or fail grade from the clinical centre involved. To date, a standardised assessment tool for evaluation of physiotherapy student performance does not exist in China.

The English APP is a practical, valid and reliable, one-page instrument that reflects standards for physiotherapy practiced in Australia [11, 12]. This instrument assesses 20 key items that reflect student competency in 7 domains (professional behaviour, communication, assessment, analysis & planning, intervention, evidence-based practice and risk management) important for physiotherapy practice. Translation of the original English APP into APP-Chinese was conducted by physiotherapy educators experienced in teaching and educational research. The original author of the APP was also involved in the translation process, to ensure the backward translated APP-Chinese version precisely matched the original English version of the APP.

The feedback from both students and CEs in our pilot study was comparable to corresponding feedback reported by Australian students and clinical educators (Tables 1 and 2). Although students and CEs were not familiar with any standardised objective assessment format for evaluation of student’s clinical performance, both groups considered the performance indicators a helpful guide for expected clinical education behaviour. Further, both the CEs and the students considered the scoring criteria appropriate and helpful. All our students agreed that the global rating score appropriately reflected their performance and over 86% of students considered the 20 item scores were a fair reflection of their performance (Table 2). This is the first study in China that reports whether students consider their performance ‘fairly’ graded.

Not surprisingly, only 66% of students agreed with the statement “The expected entry-level performance (passing level) was clear to me”. In China, physiotherapy is a developing profession and is still to be recognised as an autonomously regulated health profession by the Chinese Government. There is no legislation governing registration of the profession. In consequence the concept ‘expected entry-level performance’ was novel to both students and clinical educators in China. The percentage of agreement for the ‘clarity’ statement was higher for the CEs (Table 1) compared to the students (Table 2), probably because all CEs underwent a training workshop prior to the use of APP-Chinese and had the concept of entry-level performance explained to them. This finding also suggests that more effort should be made to educate students on the concept of ‘entry-level performance’ as a measurable standard prior to undertaking clinical placements.

A majority of the CEs expressed the view that they would prefer a smart phone application for the APP-Chinese. The use of phone applications is popular in China and many personal and official communications, calendar appointments and daily fiscal transactions are smart phone processed [21]. In the hospital or university environment, however, it not easy to implement a computerised ‘marking’ system which allows communication between students, hospital clinical educators and university academic staff, due to a strict institutional security system stipulated by the National Government. This probably explains why many of the clinical educators would prefer to use the APP-Chinese through a smart phone application.

Another advantage of using the APP-Chinese for the students in China is that this assessment tool allows a ‘*structured’* system for formative and summative feedback to be communicated to students. The importance of provision of constructive feedback targeting student strengths and areas that require improvement, were highlighted in the CEs’ APP-training workshop, prior to use of the APP-Chinese instrument. CEs were reminded that providing students with strategies to improve is an essential role of feedback. The students greatly appreciated the “strategies to improve” proposed by the CEs as this was something of a novel concept in medical education in China.

The original APP provides examples of expected student behaviour. The focus-group discussion revealed that the ‘expected behaviours’ listed in the APP-Chinese were clear and considered appropriate/practical in the clinical environment in China. However, the practicality of nurturing students’ confidence to participate and contribute at a ‘rehabilitation team meeting’ and ‘with other medical professionals’ generated discussion. Currently, many hospitals in China stipulate that interprofessional communication must only be handled by a clinical educator or senior therapist. Students are not empowered to communicate with other professions regarding patient management. Therefore, scoring for Chinese students in the expected professional behaviour domain required removal of the ‘contribution to rehabilitation team meeting and communication with medical staff’ domain from the ‘example’ list. With differences in culture and health care system in different countries, it is appropriate that students demonstrate behaviours acceptable to their specific culture and health care system. The physiotherapy program prepares students to be sensitive to cultural needs specific to patients with different cultural backgrounds, and respect and function in a health care system appropriate to a specific locale. Therefore, further research is warranted to explore how this modification of expected professional behaviour should be assessed.

### Limitations of the study

The aim of this pilot study was to explore the acceptance of a ‘standardised, structured’ assessment tool for objective evaluation of clinical performance by students and clinical educators during physiotherapy placements. For this instrument to be used confidently and extensively, a full validity and reliability study, including test-retest reliability, internal consistency, content validity, and criterion validity is required. This will be undertaken during the second phase of our study.

Another limitation of the study is the small number of students who participated. However, 15 students represented the entire cohort for the physiotherapy program at Tongji University. Qualified physiotherapy academics are limited throughout China. There are currently only six physiotherapy programs in China that have been fully accredited by WCPT [1]. Regarding staff student ratio the Sunshine Rehabilitation Centre should be commended for appointing 14 qualified physiotherapists to supervise 3 clinical placements for 15 students. It is also our plan to repeat this study with a much larger student sample and involve the CEs in all 6 accredited physiotherapy programs in China.

There are about ten other entry-level programs in China delivering a physiotherapy curriculum which follows WCPT guidelines, but none of these programs are using any structured assessment tool, let alone one that has been validated. We therefore feel it is important to encourage every physiotherapy program to adopt a proper, structured, objective assessment tool for evaluation of student clinical performance. It is our intention to introduce the validated APP-Chinese assessment instrument to all 16 physiotherapy programs in China. Our team is also in the process of integrating with the APP Linkup system in Australia, an online platform that collects student performance data from all involved universities. The database obtained from the APP Linkup system potentially allows comparison of deidentified average APP item and total scores between universities both nationally and internationally. We believe that the use of APP-Chinese by all physiotherapy programs in China will assist with the establishment of a consistent standard of professional clinical performance to ensure safe and effective physiotherapy practice in China.

## Conclusions

It is our view that the APP-Chinese assessment form is likely to be well accepted by physiotherapy students and CEs in China. However, there is still a need to evaluate the APP-Chinese on a conceptual, semantic, and content equivalence with the original source form and the internal consistency and content validity are as yet to be established. We recommend the APP-Chinese assessment tool be piloted in all 16 clinical physiotherapy programs in China as a standardised, structured, assessment tool for physiotherapy students in the clinical environment. The use of the APP-Chinese will allow future performance comparisons, not only across all programs in China, but also with international student cohorts.

## Supplementary information

**Additional file 1.**

**Additional file 2.**

## Data Availability

The datasets used and/or analysed during the current study are available from the corresponding author on reasonable request.

## Abbreviations

WCPT: World Confederation for Physical Therapy
CEs: Clinical educators
APP: Assessment of Physiotherapy Practice
PT-CPI: American physical therapist clinical performance instrument
CAF: Common Assessment Form; CEs: clinical educators
