# Supplementary material for: Acceptance of the ‘Assessment of Physiotherapy Practice (Chinese)’ as a standardised evaluation of professional competency in Chinese physiotherapy students: an observational study
Source: BMC Med Educ. 2020 Apr 9;20:108. doi: 10.1186/s12909-020-02026-3 (PMC7147022; doi:10.1186/s12909-020-02026-3)
Supplement: Supplementary file 2 — Additional file 2. [file 12909_2020_2026_MOESM2_ESM.pdf]

Appendix II

物理治疗专业临床实践评估

Assessment of Physiotherapy Practice

中国大学

实习末期
总结性评估

学生姓名

临床机构

实习日期

0 = 不常/稀少表现临床行为指标
1 = 一些临床行为指标的表现达到合格标准
2 = 大部分临床行为指标的表现达到合格标准
3 = 大部分临床行为指标的表现达到良好标准
4 = 大部分临床行为指标的表现达到优秀标准
没有评估 = 选项没有被评估
注：评分 0 或者 1 表示没有达到最低可接受的能力

专业行为 (Professional behaviour)	请只圈选一个评分 Circle one number only					
1、表现对患者权利和知情许可的理解	0	1	2	3	4	没有评估
2、表现对学习的承诺	0	1	2	3	4	没有评估
3、表现伦理、法律和文化回应性的实践	0	1	2	3	4	没有评估
4、表现团队合作能力	0	1	2	3	4	没有评估
**沟通 (Communication)**						
5、有效与适当的沟通能力-言语上/非言语上	0	1	2	3	4	没有评估
6、表现清晰精准的临床文书记录能力	0	1	2	3	4	没有评估
**评估 (Assessment)**						
7、进行恰当的以患者为中心的问诊	0	1	2	3	4	没有评估
8、选择与测量相关健康的指标及效果	0	1	2	3	4	没有评估
9、执行恰当的体格检查评估过程	0	1	2	3	4	没有评估
**分析和计划 (Analysis and planning)**						
10、正确理解评估结果	0	1	2	3	4	没有评估
11、认别并优先排序患者的问题	0	1	2	3	4	没有评估
12、制定以患者为中心且可行的近期与远期目标	0	1	2	3	4	没有评估
13、与患者合作选择合适的治疗干预	0	1	2	3	4	没有评估
**干预 (Intervention)**						
14、恰当地执行治疗干预	0	1	2	3	4	没有评估
15、是有效的教育者	0	1	2	3	4	没有评估
16、监控治疗干预效果	0	1	2	3	4	没有评估
17、恰当地推进治疗过程	0	1	2	3	4	没有评估
18、执行制定出院计划	0	1	2	3	4	没有评估
**循证实践 (Evidence-based practice)**						
19、将循证实践应用于患者为中心的服务	0	1	2	3	4	没有评估
**风险管理 (Risk management)**						
20、识别不良事件/险兆事件，最小化评估和治疗过程中的风险	0	1	2	3	4	没有评估

作为一个临床导师，您觉得您的带教学生在临床实践中的总体表现为：<br>不足够　　　　　　足够　　　　　良好　　　　　优秀
**评分规则 (Evaluation rules)**<br>√ 只在学生没有机会展现临床行为指标时才选择"没有评估"选项。<br>√ 如果选项是没有被评估，那其选项就没有得分，APP 的总分便要因缺少的选项而需相应调整<br>√ 每个选项只应圈选一个评分<br>√ 如果学生水平介于两个评分之间，以高分项计分。<br>√ 参照准入级物理治疗师最低能力的水平来评价学生的表现。

评分____/_____　　　　　　百分比______%

由*上海市养志康复医院（上海市阳光康复中心）经准许翻译。原稿参考：Dalton M et al 2011. The Assessment of Physiotherapy Practice (APP) is a valid measure of professional competence of physiotherapy students: evidence from Rasch analysis. J of Physiotherapy, 57(4):239-246. ISSN: 1836-9561*

翻译员：Translation team: HuJia[1], Alice Jones[2], Shirley Ngai[3], Joseph Siu[4]

[1] Sunshine Rehabilitation Centre, Shanghai, China

[2]Faculty of Health Sciences, University of Sydney, Australia

[3]Department of Rehabilitation Sciences, Hong Kong Polytechnic University, Hong Kong

[4]Department of Physical Therapy, University of Nebraska, USA
